# Performance Evaluation of the Deep Learning Based Convolutional Neural Network Approach for the Recognition of Chest X-Ray Images

**DOI:** 10.3389/fonc.2022.932496

**Published:** 2022-06-29

**Authors:** Sandhya Sharma, Sheifali Gupta, Deepali Gupta, Junaid Rashid, Sapna Juneja, Jungeun Kim, Mahmoud M. Elarabawy

**Affiliations:** ^1^ Chitkara University Institute of Engineering and Technology, Chitkara University, Baddi, India; ^2^ Chitkara University Institute of Engineering and Technology, Chitkara University, Rajpura, India; ^3^ Department of Computer Science and Engineering, Kongju National University, Cheonan, South Korea; ^4^ KIET Group of Institutions, Ghaziabad, India; ^5^ Department of Software, Kongju National University, Cheonan, South Korea; ^6^ Department of Computer Science, College of Computers and Information Technology, Taif University, Taif, Saudi Arabia; ^7^ Department of Mathematics, Faculty of Science, Suez Canal University, Ismailia, Egypt

**Keywords:** biomedical images, convolutional neural network, deep learning, chest X-rays, optimizers

## Abstract

Recent advancement in the field of deep learning has provided promising performance for the analysis of medical images. Every year, pneumonia is the leading cause for death of various children under the age of 5 years. Chest X-rays are the first technique that is used for the detection of pneumonia. Various deep learning and computer vision techniques can be used to determine the virus which causes pneumonia using Chest X-ray images. These days, it is possible to use Convolutional Neural Networks (CNN) for the classification and analysis of images due to the availability of a large number of datasets. In this work, a CNN model is implemented for the recognition of Chest X-ray images for the detection of Pneumonia. The model is trained on a publicly available Chest X-ray images dataset having two classes: Normal chest X-ray images and Pneumonic Chest X-ray images, where each class has 5000 Samples. 80% of the collected data is used for the purpose to train the model, and the rest for testing the model. The model is trained and validated using two optimizers: Adam and RMSprop. The maximum recognition accuracy of 98% is obtained on the validation dataset. The obtained results are further compared with the results obtained by other researchers for the recognition of biomedical images.

## 1 Introduction

Fungi, bacteria, or viruses fill the lungs with fluid, which is the main reason for the infection of the lungs. This infection which is known as Pneumonia causes effusion of the pleural as well as inflammation in the air sacs. Pneumonia is the cause of 15% of the deaths in children who are below the age of 5 years (1). Pneumonia is more prevalent in underdeveloped or developing ‘countries with scanty medical resources. This disease can be fatal if not diagnosed and treated early. It can cause the failure of the respiratory system or inflammation can be uncontrolled. For its diagnosis: Magnetic resonance imaging (MRI), radiography (X-ray), or computed tomography (CT) is frequently used, whereas radiography or X-ray is most commonly used as it is an inexpensive and non-invasive examination. Chest X-ray is the radiology test in which the chest is exposed to radiation to produce an image of the internal organs of the chest. Once the chest X-ray is produced then the radiologist interprets the organs of the chest by looking at the image produced. An automated system is required which can detect the Pneumonia for the X-ray image as manual chest X-ray examination is prone to variability (2). Sometimes the X- rays are not very clear, which makes it difficult for the radiotherapist to identify the features that help in the detection of disease. Therefore, a deep learning-based CNN model is required that can automatically recognize the healthy and pneumonic lungs from the Chest X-ray images (3). Deep learning and machine learning in the field of Artificial Intelligence that is being used in various fields like visual recognition, natural language processing (NLP), self-driving cars, fraud detection, news aggregation, and so on. Deep Learning based Models work similar to that of a human brain, by using data as the input so that it can be processed through various layers for its recognition. These models can find the hidden features that are sometimes not possible to detect by radiologists. CNN is the main tool of deep learning models which can automatically find the various features of the images which makes the recognition of images possible for different classes.

**Figure 1:**
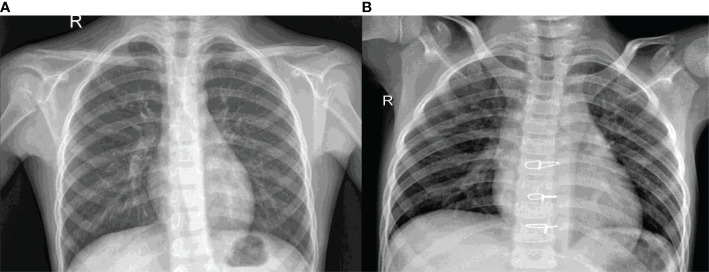
X-ray images of the lungs **(A)** Healthy chest X-ray **(B)** Pneumonic chest X-ray.

**Figure 1** shows the X-ray images of the lungs for the healthy and Pneumonic chest. **Figure 1A** is the healthy X-ray whereas **Figure 1B** is the Pneumonic X-ray. It can be seen that the pneumonic X-ray has white spots called infiltrates.

The paper is structured as: A review similar to the related work is given in Section 2; while Section 3 represents the methods used in this paper. The results obtained are depicted in Section 4 which is followed by the conclusion in Section 5. The contributions of the proposed work are given below:

• A CNN based model is developed which can be used for the recognition of chest X-ray images of the lungs.
• A model is evaluated to find the suitable optimizer, number of epochs as well as the learning rate (LR) which can work well on the available dataset.
• A Comparison of the proposed work with other developed models for the recognition of biomedical images.

## 2 Related Work

Detect Pneumonia using chest X-ray images is a problem for many years (4, 5). Sometimes the chest X-rays are not clear enough to identify the ‘features’ that help in the diagnosis of the disease. Various machine learning, as well as deep learning models, are being used for the detection and classification of pneumonia. In machine learning techniques, various features are required to be manually extracted whereas features are automatically extracted by employing deep learning techniques for detection and classification. In this section, an attempt has been made to present the work that has been done for the recognition of biomedical images using Machine Learning as well as Deep Learning techniques. Chandra et al. (6) have employed a machine learning technique to detect pneumonia. First, the lung regions are segmented from the X-ray, and later eight statistical features are extracted from the regions which are used for classification. Once the features are extracted, various classifiers are implemented for classification like the random forest, logistic regression, and multilayer perceptron (MLP) technique. The model is evaluated on 412 images and the accuracy obtained is 95.39%. Yue et al. (7) have extracted six features from the 52 CT scanned images for the detection of pneumonia. An AUC (area under the curve) of 97% is achieved. Similarly, Kuo et al. (8) have also manually extracted various features for the detection and classification of Pneumonia. The extracted features are applied to various classification models such as decision tree, support vector machine (SVM), and logistic regression. The maximum accuracy obtained is 94.5% using the decision tree.

Features are manually extracted in machine learning techniques (9, 10) whereas features are automatically extracted in deep learning techniques (11, 12). Tuncer et al. (13) has also employed a machine learning-based technique, which has further imposed the fuzzy tree transformed technique on the images. Then, various features are extracted with the help of multi-kernel local binary pattern, and later various classifiers are employed. Obtained accuracy is 97.01%. Various deep learning techniques are being employed for the detection and classification of chest X-rays.

Sharma et al. (14) implemented a CNN based model for the classification of pneumonia in Chest X-rays. The data augmentation technique is also employed to increase the data. Obtained accuracy is 90.68%. Similarly, Stephen et al. (15) also employed CNN based model by incorporating a data augmentation technique and obtained an accuracy of 93.73%. Rajpukar et al. (16) have also implemented a Deep learning-based DenseNet-121 CNN model for classifying pneumonia from Chest X-ray images. F1 score of only 76.8% is achieved. The model has not perform well due to the unavailability of the patient’s history. It has been found that various machine learning and deep learning models have been implemented for recognition. Manual extraction of features is a cumbersome task as required in machine learning models, but features are not required to be manually extracted in deep learning models, which makes them easier to implement. In this work, we have obtained a recognition accuracy of 98% using a 12 layered customized CNN model with the help of RMSprop Optimizer. A suitable optimizer has also been obtained for the available dataset. The prepared model has also been evaluated using Adam Optimizer while other parameters are kept the same.

## 3 Proposed Approach

**Figure 2:**
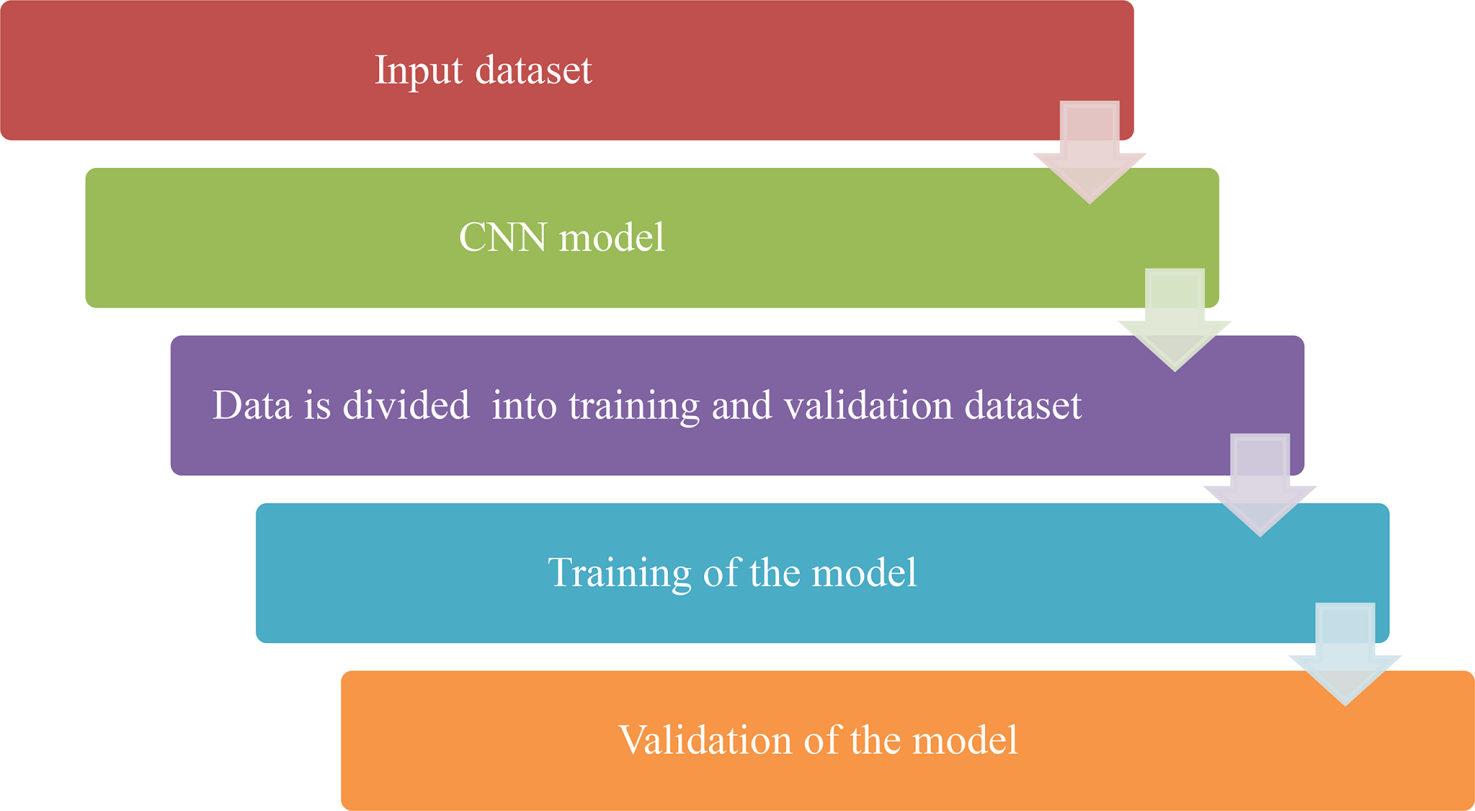
Methodology of the proposed work.

CNN based model is proposed for the recognition and classification of the Chest X-ray images. Publically available Kaggle dataset of X-ray images is used for recognition. The proposed Methodology of the work is given in **Figure 2** below. It can be seen from the figure that an X-ray image is given as input to the CNN model. First, the image is resized to 64X32 and then it will pass through some different layers (convolution, pooling, and flattening) after dividing into training and validation datasets. In the end, the obtained image will be passed through the output class which will predict the class of that image i.e. Pneumonic or Healthy image.

### 3.1 Input Dataset

**Figure 3:**
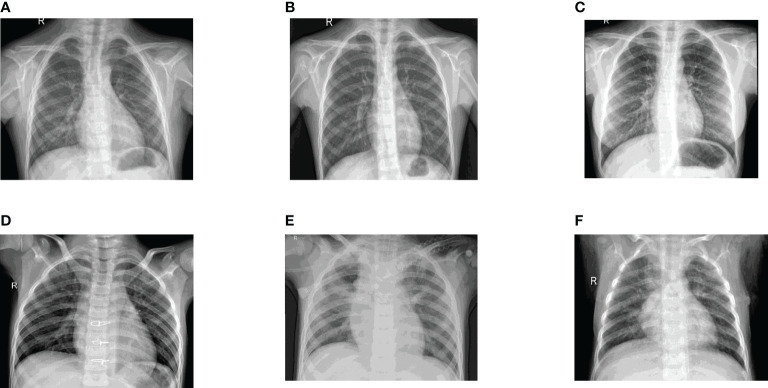
Dataset Images: **(A)** Healthy Chest X-ray 1, **(B)** Healthy Chest X-ray 2, **(C)** Healthy Chest X-ray 3, **(D)** Pneumonic Chest X-ray 1, **(E)** Pneumonic Chest X-ray 2 and **(F)** Pneumonic Chest X-ray 3.

Kaggle dataset is used for classification and recognition. Data preprocessing is not required as the dataset is already available in a clean and pre-processed format. The dataset consists of two classes of chest X-ray images where each class has 5000 images of Healthy and Pneumonic Chest X-rays. 4000 images of each class are used to train the model, and 1000 images of each class are used to test the model. Few of the dataset images for Healthy and Pneumonic Chest X-rays can be observed in **Figure 3**.

### 3.2 Data Augmentation

**Figure 4:**
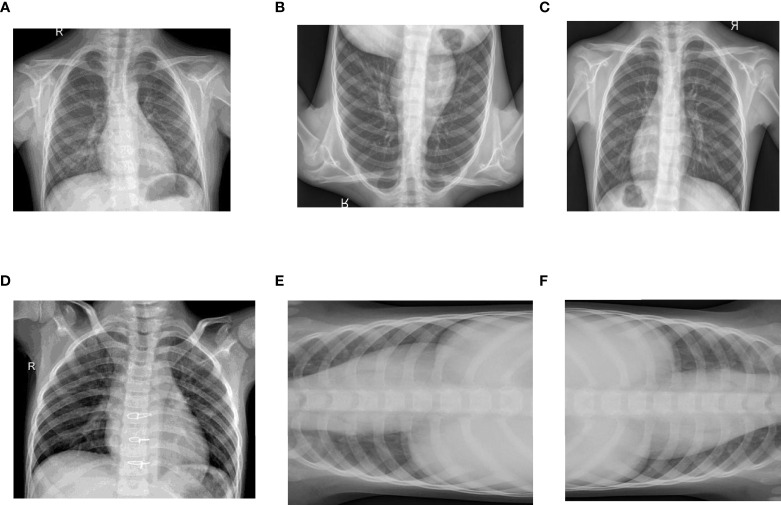
Data Augmentation of Images: **(A)** Original Healthy Chest X-ray, **(B)** Healthy Chest X-ray Flipped Vertically, **(C)** Healthy Chest X-ray Flipped Horizontally, **(D)** Original Pneumonic Chest X-ray, **(E)** Pneumonic Chest X-ray rotated 90 degrees left and **(F)** Pneumonic Chest X-ray rotated 90 degrees right.

To increase the number of images for each class, a data augmentation technique is employed. As data augmentation helps in improving the performance of the model a model is trained on different examples of the dataset. Flipping and Rotation of the existing dataset are carried out to enhance the dataset. **Figure 4** is showing the flipped and rotated images of the dataset.

### 3.3 Proposed Convolution Model

A total of 10000 images of healthy and pneumonic chest X-rays have been taken and each class has 5000 samples.

**Figure 5:**
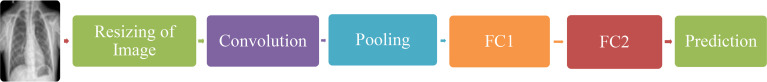
Block diagram of the CNN Model.

**Figure 6:**
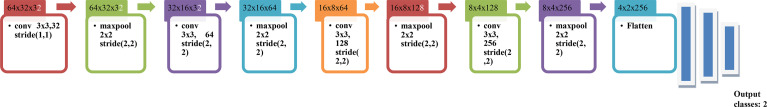
Layered Architecture of the CNN Model.

All the samples are passed through the set of three main layers of the CNN model: convolution, pooling and then the fully connected layer. The basic block diagram of the proposed model is shown in **Figure 5**. CNN model has been the biggest contributor in the field of image analysis and computer vision. CNN model or ConvNet can learn the features of various images which are used for their classification and recognition. The convolution layer helps in obtaining the feature maps from the images which are the extraction of various features. Automatic feature extraction takes place using a convolution layer. The pooling layer helps in retaining the important features while it also helps in dropping off the features which are least important for the recognition. This layer helps reduce the image size. The fully connected layer at the end helps in obtaining the output as classes. The architecture of the CNN model is shown in **Figure 6**. The X-ray image is given as input to the first layer of the model, i.e. convolution layer with 32 filters and the size of each filter is 3x3 which will help in obtaining 32 feature maps. These are given to the next layer: the Maxpooling layer with a filter pool of size 2x2 and a stride with the size (2, 2). All Maxpooling layers used in the model have the same size. In the next convolution layer, the number of filters is increased from 32 to 64 with the same size 3x3 as used earlier, which is again followed by a max-pooling layer. The number of filters in the next convolution layer increases to 128 which is followed by the max-pooling layer, and the last convolution layer has 256 filters (17, 18). In the end, a flattening layer is introduced so that the obtained pooled feature maps can be fed to the array of the neural network (19, 20). Two dense layers are introduced after the flattening layer which is followed by an output layer.

## 4 Results and Analysis

In this section, results obtained for the recognition of chest X-rays are shown. The CNN model is trained using 80% of the data for the recognition of healthy and pneumonic chest X-rays. Once the model is trained, then it is validated using the rest of the images. The model is trained using RMSprop as well as Adam optimizer with an LR of 0.01 and the number of epochs used is 30. The model has not perform well with the other optimizers as compared to the two selected optimizers. Even, when the number of epochs are increased from 30, the model’s performance started to deteriorate or either remained the same. Therefore, the model performed at its best with the selected optimization parameters. All the results are obtained on the Google Colab, which is a free Jupyter environment that runs on the cloud.

### 4.1 Result Analysis Using RMSprop Optimizer

The model has been trained and validated with the help of RMSprop Optimizer where the number of epochs using which the model is trained is 30, LR used is 0.01 and Batch size of 4 is used. The model is validated with the help of various parameters such as accuracy, loss, recall, area under the curve (AUC) and precision (19, 21).

#### 4.1.1 nTraining and Validation: Accuracy and Loss Curves

**Figure 7:**
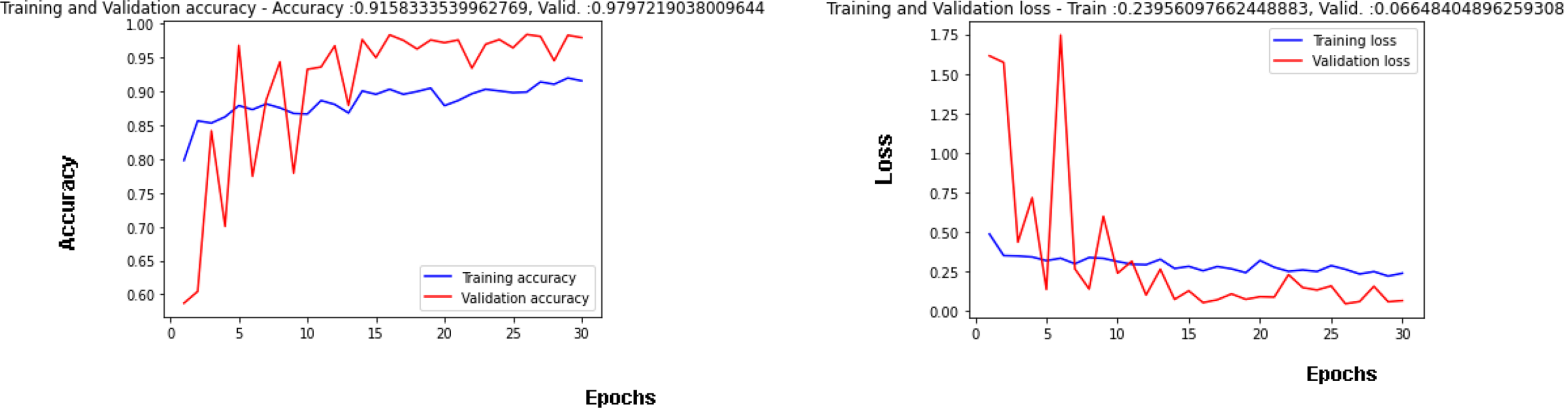
Training and Validation Curves using RMSprop Optimizer: **(A)** Accuracy, **(B)** Loss.

**Figure 7** is showing the results obtained on the training as well as the validation dataset. A maximum validation accuracy of 98% is obtained on the 29^th^ epoch. In **Figure 7A**, Validation accuracy (22) is below 60% on the 1^st^ epoch and increases till 3^rd^ epoch to around 84% and then decreases on 4^th^ epoch and again increases on the next epoch (23–25). The highest validation accuracy obtained is 98% on the 29th epoch, and it is 97% on the last epoch. Training accuracy also started at around 80% in the 1^st^ epoch and going close to 90% in the last epoch. **Figure 7B** is showing the curve for the training and validation dataset. Validation loss is around 1.61 in the 1^st^ epoch and is decreasing till the 5^th^ epoch to 0.13 but again increasing in the 6^th^ epoch to 1.74. The value obtained for validation loss is 0.06 on the last epoch but the minimum validation loss obtained is 0.05 on the 29^th^ epoch itself. Training loss is 0.50 in the 1^st^ epoch and decreases to close to a value of 0.30 in the last epoch.

#### 4.1.2 Confusion Matrix Parameters Analysis

Performance metrics are used for the recognition of patterns and the classification of images. These parameters are important in building a perfect model which can give more accurate and precise results. Maximum validation precision, maximum validation recall, and maximum validation AUC obtained are 0.05, 98%, 98% and 99% on the 29th epoch.

**Figure 8:**
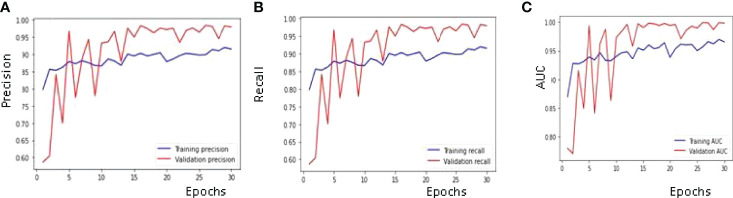
Confusion Matrix Parameter Curves using RMSprop Optimizer: **(A)** Precision, **(B)** Recall and **(C)** AUC.

The best results for the available dataset using the proposed CNN model are obtained on the 29^th^ Epoch. **Figure 8A** shows the curve of precision for the training and validation dataset. The value of precision for the validation data is below 0.60 on the 1^st^ epoch, increasing till the 3^rd^ epoch and later decreasing and then increasing. The maximum value of the precision is 0.98 on the 29^th^ epoch, while 0.97 on the last epoch. The value of precision for the training dataset is around 0.80 on the 1^st^ epoch and goes close to 0.90 on the last epoch. The parameter convergence plot for Recall is shown in **Figure 8B**. Recall for the validation dataset is below 0.60 on the 1^st^ epoch and 0.97 on the last epoch. The maximum value for the recall is 0.98 on the 29^th^ epoch. The value of recall for the training dataset is 0.80 on the 1^st^ epoch and close to 0.90 on the last epoch. The AUC for the validation and training dataset is shown in **Figure 8C**. The highest value of AUC achieved is around 0.99 on the 29^th^ as well as 30^th^ epoch, while the minimum value of AUC is 0.77 on the 2^nd^ epoch and it started at 0.78 on the 1^st^ epoch. The AUC for the training data is around 0.87 on the 1^st^ epoch and is going close to 0.96 on the 30^th^ epoch.

### 4.2 Result Analysis Using Adam Optimizer

Here the model is trained and validated using Adam Optimizer (19, 20). The number of epochs using which the model is trained is 30, the LR used is 0.01, and a batch size of 4 is used. The model is validated with the help of various parameters like accuracy, loss, Recall, Area under the curve (AUC) (19, 26), and precision (22, 27).

#### 4.2.1 Training and Validation: Accuracy and Loss Curves

**Figure 9:**
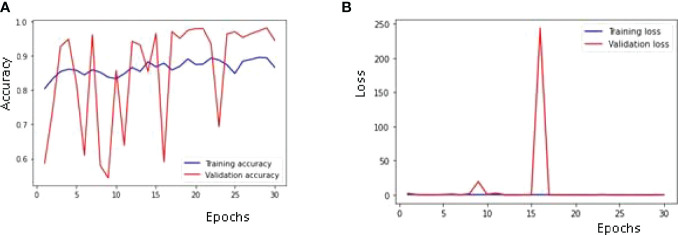
Training and Validation Curves using Adam Optimizer: **(A)** Accuracy, **(B)** Loss.

**Figure 9** is showing the results obtained on the training as well as the Validation dataset. The maximum validation accuracy of 97% is obtained on the 29^th^ epoch. It can be observed from **Figure 9A** that the validation accuracy is below 60% in the 1^st^ epoch and increases till the 4^th^ epoch to around 94% and then decreases in the 5^th^ and 6^th^ epoch and again increases in the next epoch. The highest validation accuracy obtained is 97% on the 29th epoch and it is 94% on the last epoch. Training accuracy also started at around 80% in the 1^st^ epoch and is below 90% till the last epoch. **Figure 9B** is showing the loss curve for the training and validation dataset. The validation loss is around 2.2 in the 1^st^ epoch and is decreasing till the 5^th^ epoch to 0.47 but again increasing in the 6^th^ epoch to 1.25. The value obtained for validation loss is 0.15 on the last epoch but the minimum validation loss obtained is 0.08 on the 29^th^ epoch itself. Training loss is around 2 in the 1^st^ epoch and decreases to close to a value of 0.80 in the last epoch.

#### 4.2.2 Confusion Matrix Parameters Analysis

**Figure 10:**
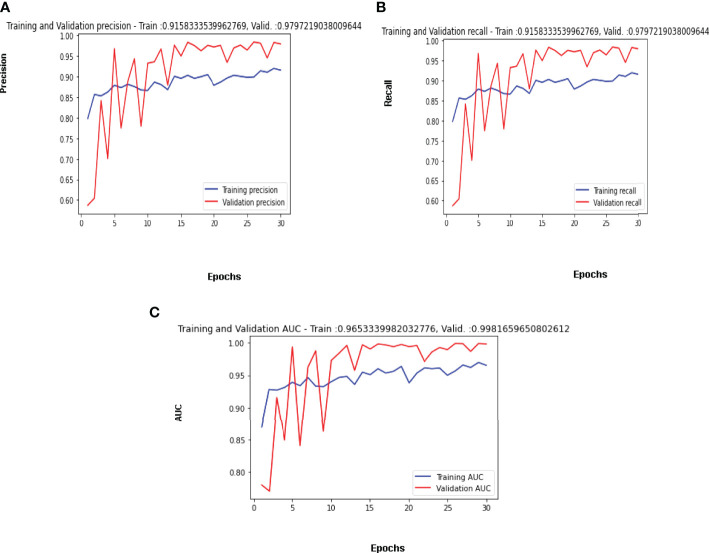
Confusion Matrix Parameter Curves Using Adam Optimizer: **(A)** Precision, **(B)** Recall, and **(C)** AUC.

Maximum validation precision, maximum validation recall, and maximum validation AUC obtained are 98%, 98% and 99% on the 29^th^ epoch. The best results for the available dataset using the proposed CNN model are obtained on the 29^th^ Epoch. **Figure 10A** is shows the curve of precision for the training and validation dataset. The value of precision for the validation data is below 0.60 on the 1^st^ epoch, increasing till the 5th epoch and later decreasing and then increasing. The maximum value of the precision is 0.98 on the 29^th^ epoch, while 0.94 on the last epoch. The value of precision for the training dataset is around 0.80 on the 1^st^ epoch and goes close to 0.87 on the last epoch. The parameter convergence plot for Recall is shown in **Figure 10B**. Recall for the validation dataset is below 0.60 on the 1^st^ epoch and 0.94 on the last epoch. The maximum value for the recall is 0.98 on the 29^th^ epoch. The value of recall for the training dataset is 0.80 on the 1^st^ epoch and close to 0.87 on the last epoch. The AUC for the validation and training dataset is shown in **Figure 10C**. The highest value of AUC achieved is around 0.99 on the 29^th^ and it is 0.98 on the 30^th^ epoch while the minimum value of AUC is 0.67 on the 1st epoch. The AUC for the training data is around 0.87 on the 1^st^ epoch and is going close to 0.93 on the 30^th^ epoch.

### 4.3 Results Comparison of Adam and RMS Optimizer

**Figure 11:**
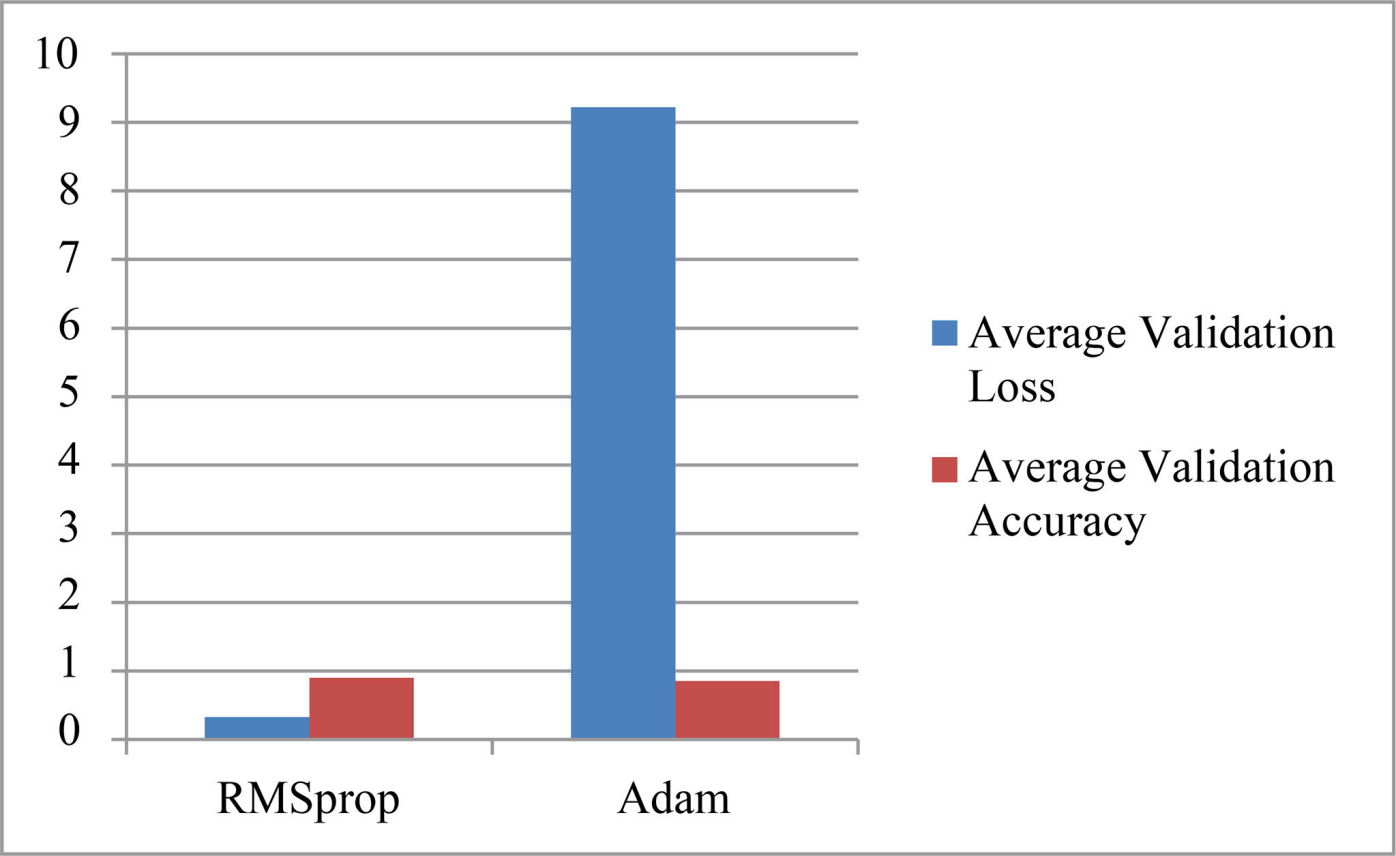
Average Validation Values Obtained by using two Optimizer.

The parameter values obtained by using two optimizers: RMSprop and Adam are shown in **Table 1**. The table shows the results for various validation parameters such as accuracy, loss, precision, recall, and AUC (25). A model is trained and validated using 30 epochs, it can be seen that the maximum accuracy is obtained on the 29^th^ epoch using both the optimizers. RMSprop has achieved the maximum accuracy of 98% with a minimum loss of 0.05 while Adam optimizer has obtained the maximum accuracy of 97% with a minimum loss of 0.08. The results for the average accuracy of the validation and loss are also depicted in **Table 2**. Average Validation loss and Accuracy using RMSprop optimizer is 0.33 and 90% while 9.22 and 85% using Adam Optimizer. The graph representation for the same is given in **Figure 11**.

**Table 1:** Results obtained on the Validation dataset using two optimizers: RMSprop and Adam.

Optimizer	Epoch	Accuracy	Loss	Precision		Recall	AUC
	1	0.58	1.61	0.58		0.58	0.78
	2	0.60	1.57	0.60		0.60	0.77
	3	0.84	0.43	0.84		0.84	0.91
	4	0.70	0.71	0.70		0.70	0.84
	5	0.96	0.13	0.96		0.96	0.99
RMSprop	6	0.77	1.74	0.77		0.77	0.84
	7	0.88	0.26	0.88		0.88	0.96
	28	0.94	0.15	0.94		0.94	0.98
	29	0.98	0.05	0.98		0.98	0.99
	30	0.97	0.06	0.97		0.97	0.99
	1	0.58	2.29	0.58		0.58	0.67
	2	0.73	0.50	0.73		0.73	0.87
	3	0.92	0.25	0.92		0.92	0.97
	4	0.94	0.20	0.94		0.94	0.98
Adam	5	0.81	0.47	0.81		0.81	0.89
	6	0.61	1.25	0.61		0.61	0.81
	7	0.96	0.10	0.96		0.96	0.99
	28	0.97	0.09	0.97		0.9722	0.9944
	29	0.97	0.08	0.98		0.9815	0.9953
	30	0.94	0.15	0.94		0.9450	0.9883

**Table 2:** Comparison of the average validation loss and accuracy obtained by two optimizers.

Parameter/Optimizer	RMSprop	Adam
Average Validation Loss	0.33	9.22
Average Validation Accuracy	0.90	0.85

### 4.4 Comparison of the Proposed Work With the Existing Models

**Table 3** shows a comparison of the proposed work with existing models which have been used for the recognition of biomedical images. The table shows the values of parameters obtained for the recognition of biomedical images. It can be observed in the table that the highest recognition accuracy obtained by the proposed work is 98% by employing CNN based deep learning model in which automatic feature extraction takes place while others have achieved less accuracy.

**Table 3:** Comparison of the proposed work with existing models.

[Ref No.]/Year	Worked on	Technique used	Parameters obtained
(6)/2020	Lungs Chest X-ray	Manual feature extraction Classifiers used: Random forest, logistic regression, Multilayer perceptron (MLP) technique	Accuracy: 95.39%
(7)/2020	CT Scan Images	Manual feature extraction Classifiers used: Random forest and logistic regression.	AUC:97%
(8)/2019	Chest X-ray	Manual feature extraction Classifiers used: Decision tree, Support vector machine (SVM), and logistic regression.	Accuracy: 94.5%
(13)/2021	Chest X-ray	Manual feature extraction by employing multi-kernel local binary Machine Learning based classifier	Accuracy: 97.01%.
(14)/2020	Chest X-ray	Automatic Feature Extraction Deep Learning based CNN Model	Accuracy: 90.68%.
(15)/2019	Chest X-ray	Automatic Feature Extraction Deep Learning based CNN Model	Accuracy: 93.73%.
(16)/2017	Chest X-ray	Automatic Feature Extraction DenseNet-121 CNN model	F1 Score: 76.8%
Proposed Model	Chest X-ray	Automatic Feature Extraction Deep Learning based CNN Model	Accuracy:98%

## 5 Conclusion

As Pneumonia is a life-threatening disease that occurs due to an infection in the lungs if not detected at an early stage. Chest X-ray is the most common method for the diagnosis of the disease. Radiotherapist manually observes the features from the Chest X-ray which can help in the identification of the Pneumonia. A CNN model is proposed for the recognition of chest X-rays which are classified into 2 classes: Healthy X-rays and pneumonic X-rays. A CNN model employing two optimizers: RMSprop and Adam are used for recognition. Kaggle dataset is used for the recognition having 5000 samples of images for each class. From the obtained results, it has been observed that the RMS prop optimizer with an LR of 0.01 has helped in obtaining the maximum validation accuracy of 98% while the Adam optimizer has achieved the maximum validation accuracy of 97%. In terms of the average validation values obtained using two optimizers, it has been found that the highest average accuracy and the lowest average loss of 90% and 0.33 are also achieved using RMSprop Optimizer for the available dataset. Huge data are being taken into consideration for recognition and classification. The future direction could be to think about reducing the processing time of the data. Selective information processing is the solution, which means processing the required part of the image only instead of the whole image.

It can be concluded from the obtained results that RMSprop has outperformed Adam Optimizer for the recognition of the available dataset.

## Data Availability Statement

The original contributions presented in the study are included in the article/supplementary material. Further inquiries can be directed to the corresponding authors.

## Author Contributions

Conceptualization, SS, SG, DG. Data curation, SS, SG, DG, JR, SJ, JK, ME. Formal analysis, SS, SG, DG, JR, SJ. Funding acquisition, JK and JR. Investigation, SS, SG, DG, JR, SJ. Methodology, SS, SG, DG, JR, SJ, JK. Project administration, SG and DG. Writing—original draft, SS, SG, DG, JR, SJ. Writing—review and editing, SS, SG, DG, JR, SJ, JK, ME. All authors contributed to the article and approved the submitted version.

## Funding

This research was partly supported by the Technology Development Program of MSS [No. S3033853] and by the National Research Foundation of Korea (NRF) grant funded by the Korea government (MSIT) (No. 2021R1A4A1031509).

## Conflict of Interest

The authors declare that the research was conducted in the absence of any commercial or financial relationships that could be construed as a potential conflict of interest.

## Publisher’s Note

All claims expressed in this article are solely those of the authors and do not necessarily represent those of their affiliated organizations, or those of the publisher, the editors and the reviewers. Any product that may be evaluated in this article, or claim that may be made by its manufacturer, is not guaranteed or endorsed by the publisher.
